# Supplementary material for: DeepSleep convolutional neural network allows accurate and fast detection of sleep arousal
Source: Commun Biol. 2021 Jan 4;4:18. doi: 10.1038/s42003-020-01542-8 (PMC7782826; doi:10.1038/s42003-020-01542-8)
Supplement: Supplementary file 2 — Supplementary Information [file 42003_2020_1542_MOESM2_ESM.pdf]

Supplementary Information

DeepSleep convolutional neural network allows accurate and fast detection of sleep arousal

Hongyang Li[1], Yuanfang Guan[1,*]

1. Department of Computational Medicine and Bioinformatics, University of Michigan, 100 Washtenaw Avenue, Ann Arbor, MI 48109, USA

* Corresponding author: gyuanfan@umich.edu

The system configuration to test DeepSleep runtimes

CPU

Architecture:	x86_64
CPU op-mode(s):	32-bit, 64-bit
Byte Order:	Little Endian
CPU(s):	8
On-line CPU(s) list:	0-7
Thread(s) per core:	2
Core(s) per socket:	4
Socket(s):	1
NUMA node(s):	1
Vendor ID:	GenuineIntel
CPU family:	6
Model:	94
Model name:	Intel(R) Core(TM) i7-6700K CPU @ 4.00GHz
Stepping:	3
CPU MHz:	4000.000
BogoMIPS:	8015.88
Virtualization:	VT-x
L1d cache:	32K
L1i cache:	32K
L2 cache:	256K
L3 cache:	8192K
NUMA node0 CPU(s):	0-7

GPU

NVIDIA GeForce GTX TITAN X

Memory

31GB in total

System

Linux version 4.4.16-1.el7.elrepo.x86_64 (mockbuild@Build64R7) (gcc version 4.8.5 20150623 (Red Hat 4.8.5-4) (GCC)) #1 SMP Wed Jul 27 15:27:40 EDT 2016

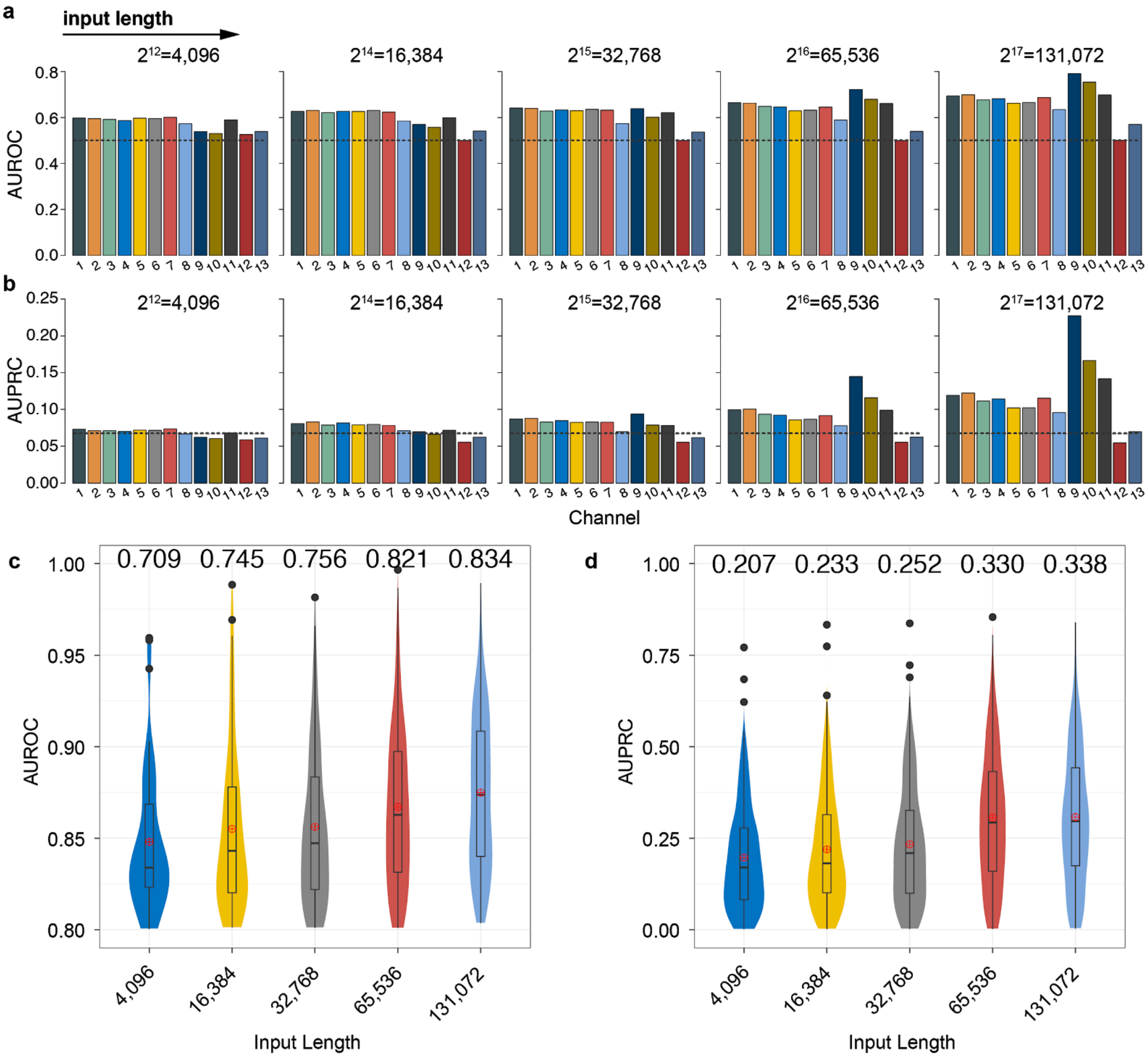

Supplementary Figure 1. The prediction performances of models using various lengths of polysomnographic recordings as input.

The (**a**) AUROCs and (**b**) AUPRCs of models using different lengths of polysomnographic recordings as input. From left to right, the length of input gradually increases from 4,096 (about 20 seconds) to 131,072 (about 11 minutes). Each color represents a model using one of the 13 polysomnographic signals. These signals correspond to the 13 channels from top to bottom in **Figure 1 - "Data"**: 1. F3-M2; 2. F4-M1; 3. C3-M2; 4. C4-M1; 5. O1-M2; 6. O2-M1; 7. E1-M2; 8. Chin; 9. ABD; 10. Chest; 11. Airflow; 12. SaO_2; 13. ECG. The dashed lines represent the baseline of random predictions in the AUROC space (baseline=0.500) and the AUPRC space (baseline=0.072). In contrast to (**a**) and (**b**) where a single channel was used as input, all 13 channels were used together as input features in (**c**) and (**d**). Longer input lengths achieved higher AUPRCs and AUROCs. The value above each violin is the overall AUPRC/AUROC, which is different from the simple mean or median value. The overall

AUPRC/AUROC considers the length of each record and longer records contribute more to the overall AUPRC/AUROC (see details in Methods - Overall AUPRC and AUROC).

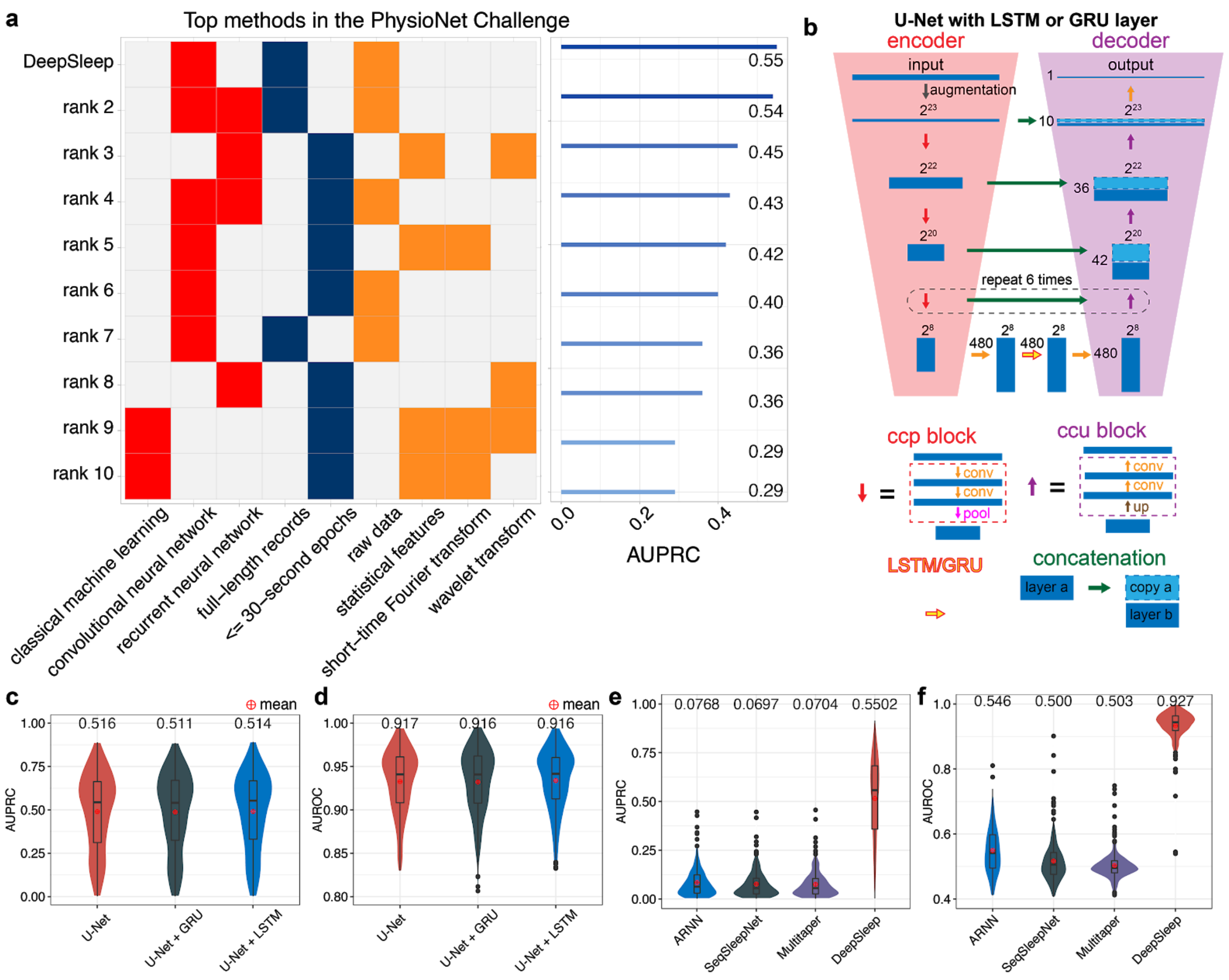

Supplementary Figure 2. The comparison of top 10 teams in the 2018 PhysioNet Challenge, recurrent neural network, and sleep staging methods.

(**a**) In the left panel, top methods, rank 2 [1], rank 3 [2], rank 4 [3], rank 5 [4], rank 6 [5], rank 7 [6], rank 8 [7], rank 9 [8], rank 10 [9] are compared in terms of machine learning models (red blocks), input length for models (blue blocks), and the types of input (orange blocks). In particular, the inputs are either raw polysomnogram data, or features extracted by statistical analysis, short-time Fourier transform, or wavelet transform. The corresponding prediction performances of these methods are shown in the right panel. We also implemented the recurrent neural network (RNN) structure by adding a recurrent unit of LSTM or GRU layer (yellow arrow with red border) at the bottom of U-Net (**b**). The arrows in different colors represent different neural network layers, blocks or operations. The prediction (**c**) AUPRCs and (**d**) AUROCs of U-Net, U-Net with GRU and U-Net with LSTM are shown in different colors. Adding the recurrent layer

did not improve the performance. We used U-Net without recurrent layers as in our final model. We further compared current methods for sleep staging. The prediction (**e**) AUPRCs and (**f**) AUROCs of (a) attention recurrent neural network (ARNN) [10], (b) SeqSleepNet using features from short-time Fourier transform [11,12], (c) a method using features from Thomson's multitaper [13,14], and (d) our DeepSleep approach are shown in different colors. The value above each violin is the overall AUPRC/AUROC, which is different from the simple mean or median value. The overall AUPRC/AUROC considers the length of each record and longer records contribute more to the overall AUPRC/AUROC (see details in Methods - Overall AUPRC and AUROC).

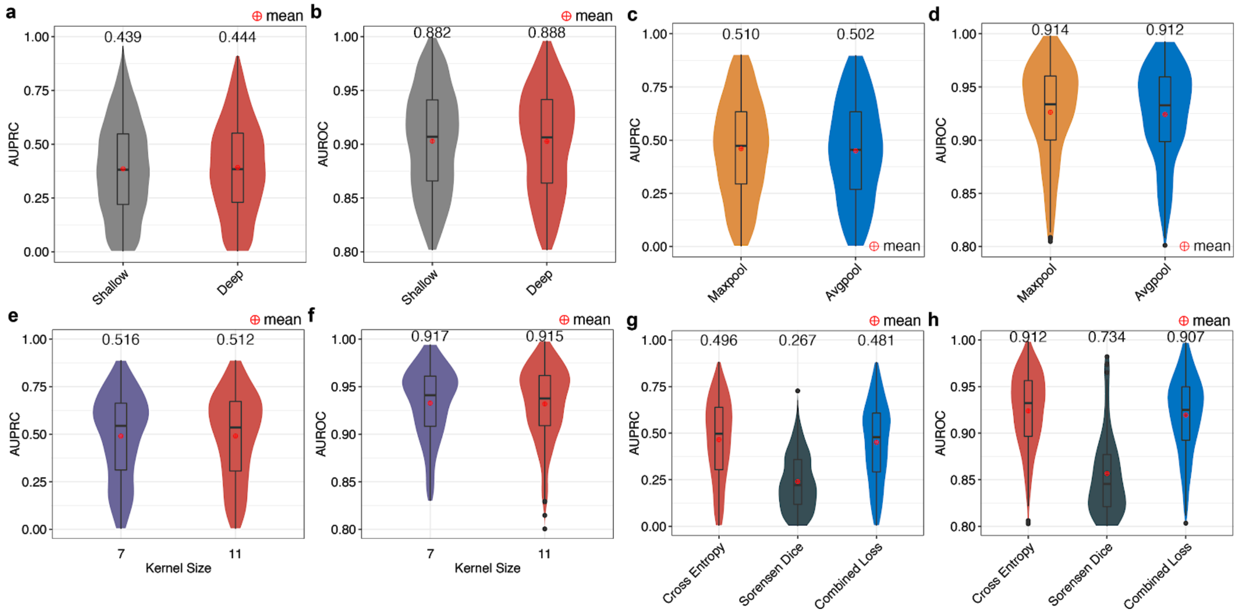

Supplementary Figure 3. The performance comparison of U-Net with different modifications.

The prediction (**a**) AUPRCs and (**b**) AUROCs of the "Shallow" and "Deep" U-Net were compared. The "Shallow" structure is only relatively shallow (4 less convolutional layers), compared with the "Deep" structure. Nevertheless, the "Shallow" U-Net already showed worse prediction performance than the "Deep" one. The prediction (**c**) AUPRCs and (**d**) AUROCs of U-Net with the kernel size of 7 and 11 in the convolutional layers were compared. Since the performances were very similar and the kernel size of 11 required more computational time and sources, we used the kernel size of 7 in our model. The prediction (**e**) AUPRCs and (**f**) AUROCs of U-Net with max-pooling or average-pooling layers are also compared. Using max-pooling layers has slightly higher performance, which was implemented in our model. The prediction (**g**) AUPRCs and (**h**) AUROCs of models trained with the cross-entropy loss, the Sorensen dice loss or combining both losses were further tested. The cross-entropy loss significantly outperformed the sorensen dice loss. Even if we combined both losses, the performance was still lower. Therefore, we used the cross-entropy loss function to train our model. The value above each model is the overall AUPRC/AUROC, which is different from the simple mean or median value. The overall

AUPRC/AUROC considers the length of each record and longer records contribute more to the overall AUPRC/AUROC (see details in Methods - Overall AUPRC and AUROC).

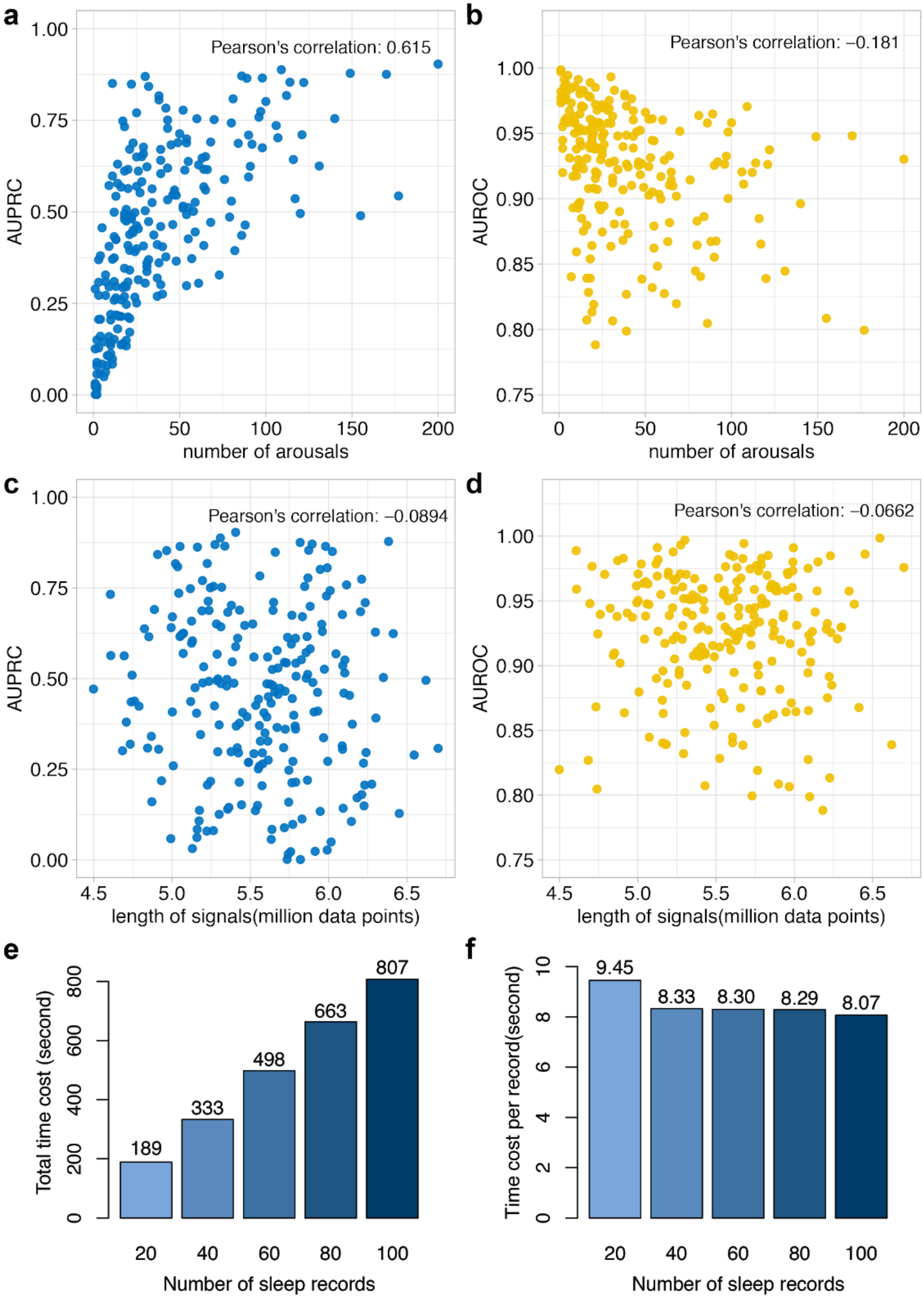
a
Pearson's correlation: 0.615
AUPRC
number of arousals
b
Pearson's correlation: −0.181
AUROC
number of arousals
c
Pearson's correlation: −0.0894
AUPRC
length of signals(million data points)
d
Pearson's correlation: −0.0662
AUROC
length of signals(million data points)
e
Total time cost (second)
189
333
498
663
807
Number of sleep records
f
Time cost per record(second)
9.45
8.33
8.30
8.29
8.07
Number of sleep records

Supplementary Figure 4. The relationship between prediction performance and the number of arousals, and the runtimes for predicting sleep arousals.

The prediction (**a**) AUPRCs and (**b**) AUROCs are shown by the y-axis. Each dot represents one sleep record. The AUPRC has a medium correlation with the number of sleep arousals. The relationships between (**c**) AUPRCs / (**d**) AUROCs and the total length of sleep record are also shown as scatter plots.
The (**e**) total time cost and (**f**) average time cost per sleep record are shown in bar plots. Notably, the average runtime per sleep record is less than 10 seconds and gradually decreases as the total number of records to be analyzed increases. This results from the overhead time of loading the large neural network models before the prediction step.

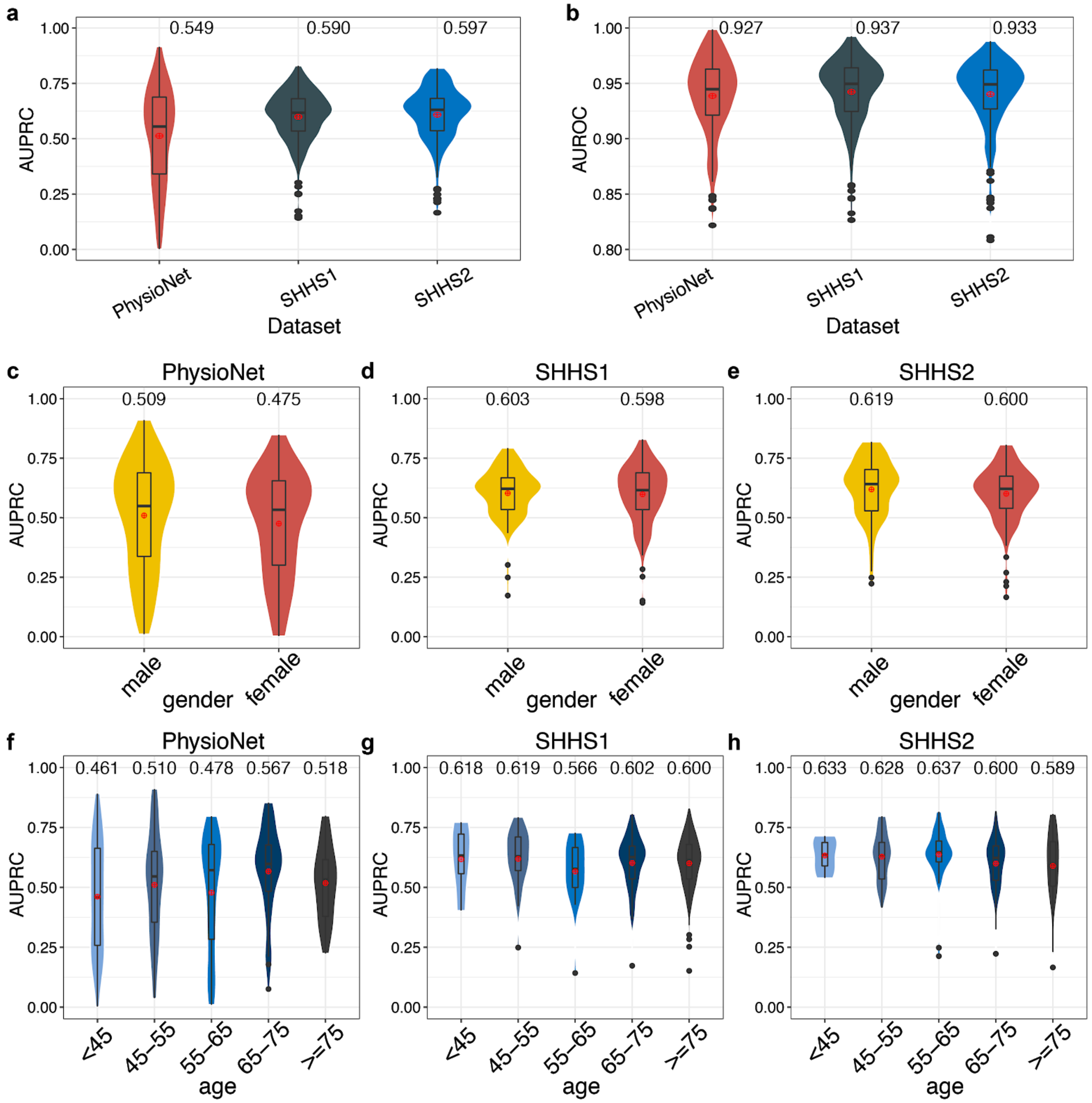

Supplementary Figure 5. The performance comparison of DeepSleep on three different datasets and different groups of individuals.

The prediction (**a**) AUPRCs and (**b**) AUROCs of DeepSleep on the 2018-PhysioNet, Sleep Heart Health Study visit 1 (SHHS1), and Sleep Heart Health Study visit 2 (SHHS2) datasets were compared. The performance on these three datasets was comparable. The performance of different groups are shown in (**c-e**) gender and (**f-h**) age in three datasets.

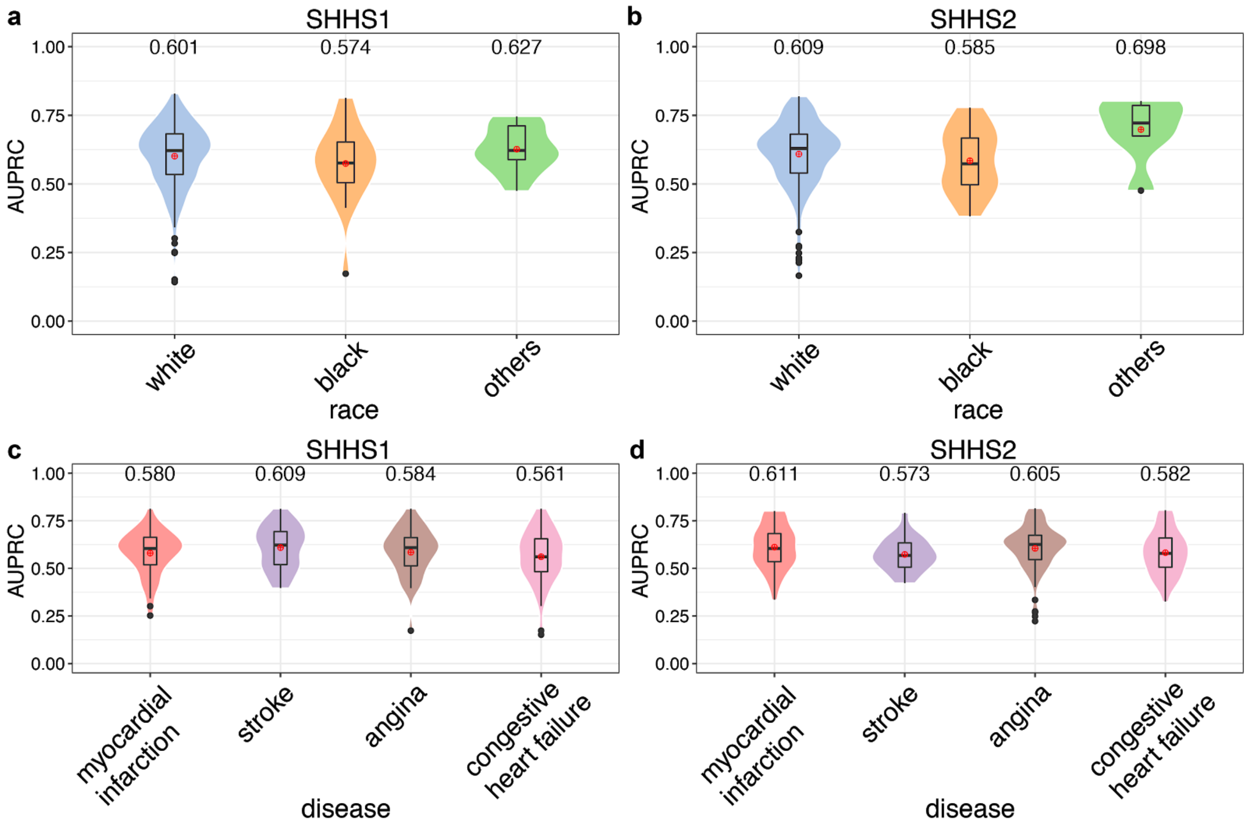

Supplementary Figure 6. The performance comparison of DeepSleep on individuals of different races and cardiovascular conditions.

The prediction AUPRCs of DeepSleep on (**a-b**) patients of different races and (**c-d**) patients with different cardiovascular conditions in the SHHS1 and SHHS2 datasets.

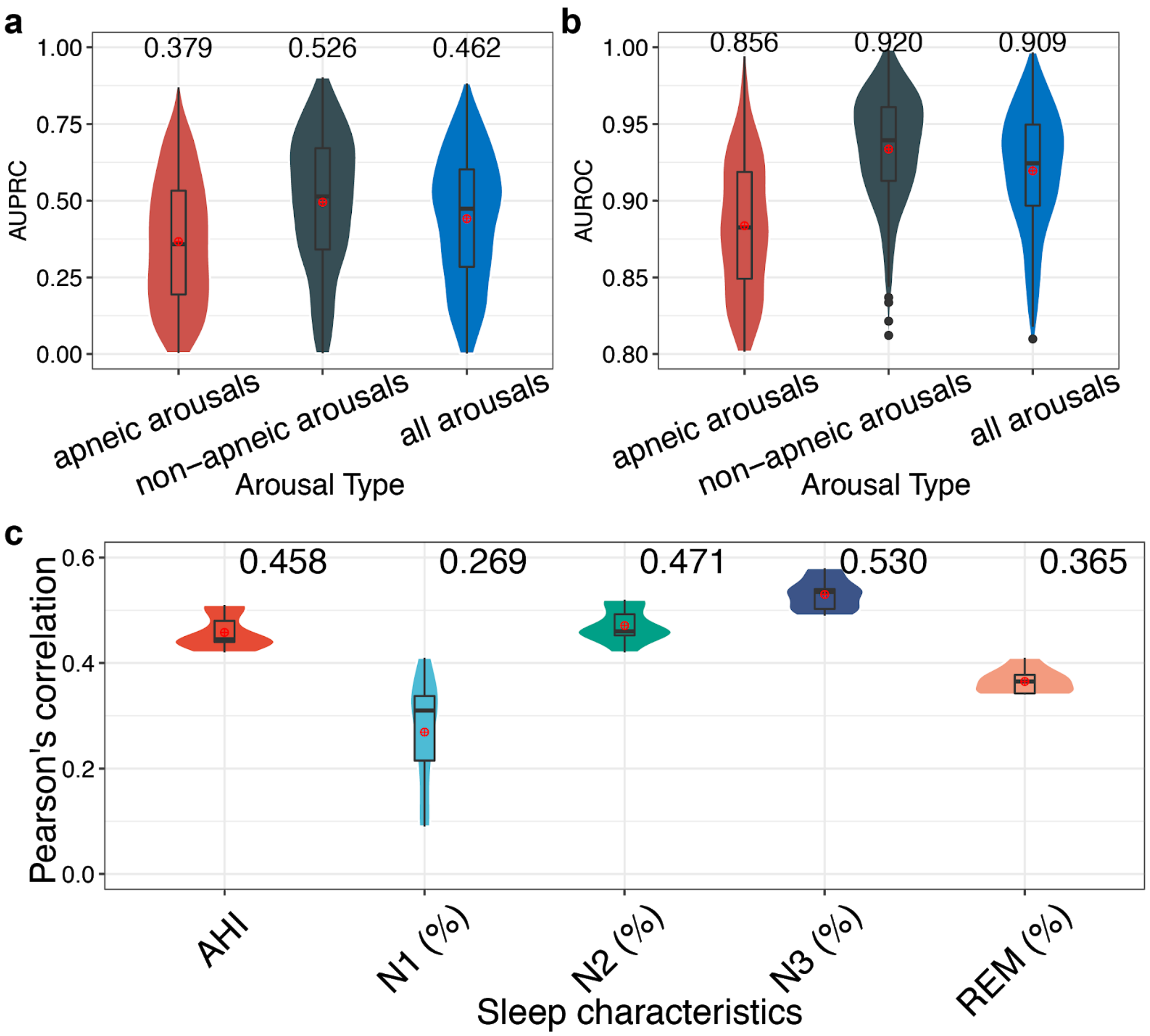

Supplementary Figure 7. The predictive performance of DeepSleep in sleep staging and scoring.

We tested the prediction (**a**) AUPRCs and (**b**) AUROCs of DeepSleep on apneic, non-apneic, and all (both apneic and non-apneic) arousals. The value above each violin is the overall AUPRC/AUROC, which is different from the simple mean or median value. Other sleep characteristics, including apnea-hypopnea index (AHI) and percentages of N1/N2/N3/REM were also predicted using the lightGBM model based on the feature maps after the encoder blocks. The Pearson's correlations between predictions and observations are shown in (**c**).

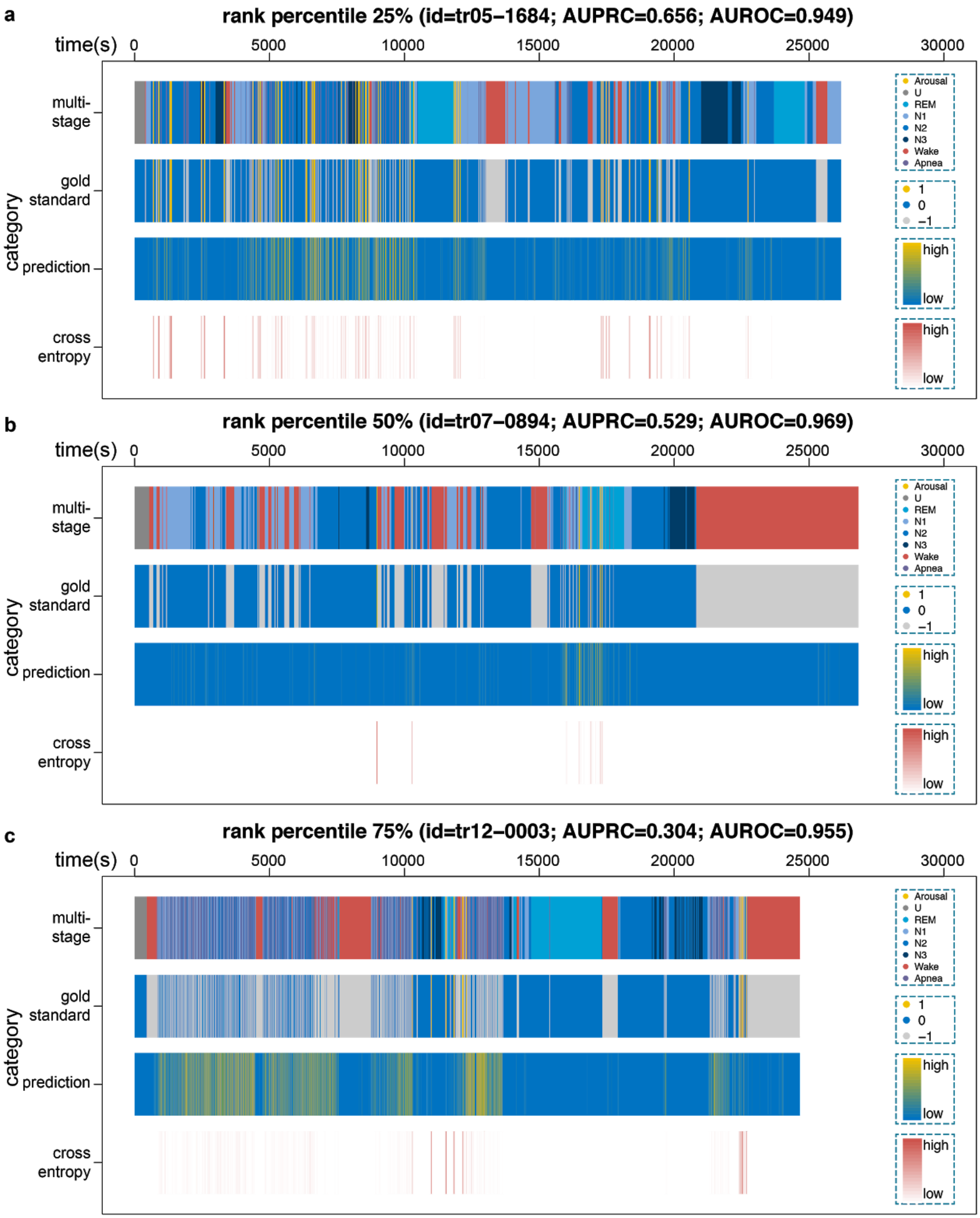

a
rank percentile 25% (id=tr05−1684; AUPRC=0.656; AUROC=0.949)
b
rank percentile 50% (id=tr07−0894; AUPRC=0.529; AUROC=0.969)
c
rank percentile 75% (id=tr12−0003; AUPRC=0.304; AUROC=0.955)
time(s)
0
5000
10000
15000
20000
25000
30000
category
multi-stage
gold standard
prediction
cross entropy
Arousal
U
REM
N1
N2
N3
Wake
Apnea
1
0
−1
high
low

Supplementary Figure 8. Visualization of our prediction and the gold standard annotation for three sleep records with rank percentile 25%, 50%, and 75% based on the prediction AUPRC.

From top to bottom along the y-axis, the four rows correspond to the 8 annotation categories, the binary label of arousal (yellow) and sleep (blue), excluding the non-scoring regions (gray), the continuous prediction and the cross entropy loss at each data point. The sleep records in (**a**), (**b**), and (**c**) were ranked 25%, 50%, and 75% respectively among all records based on the prediction AUPRC.

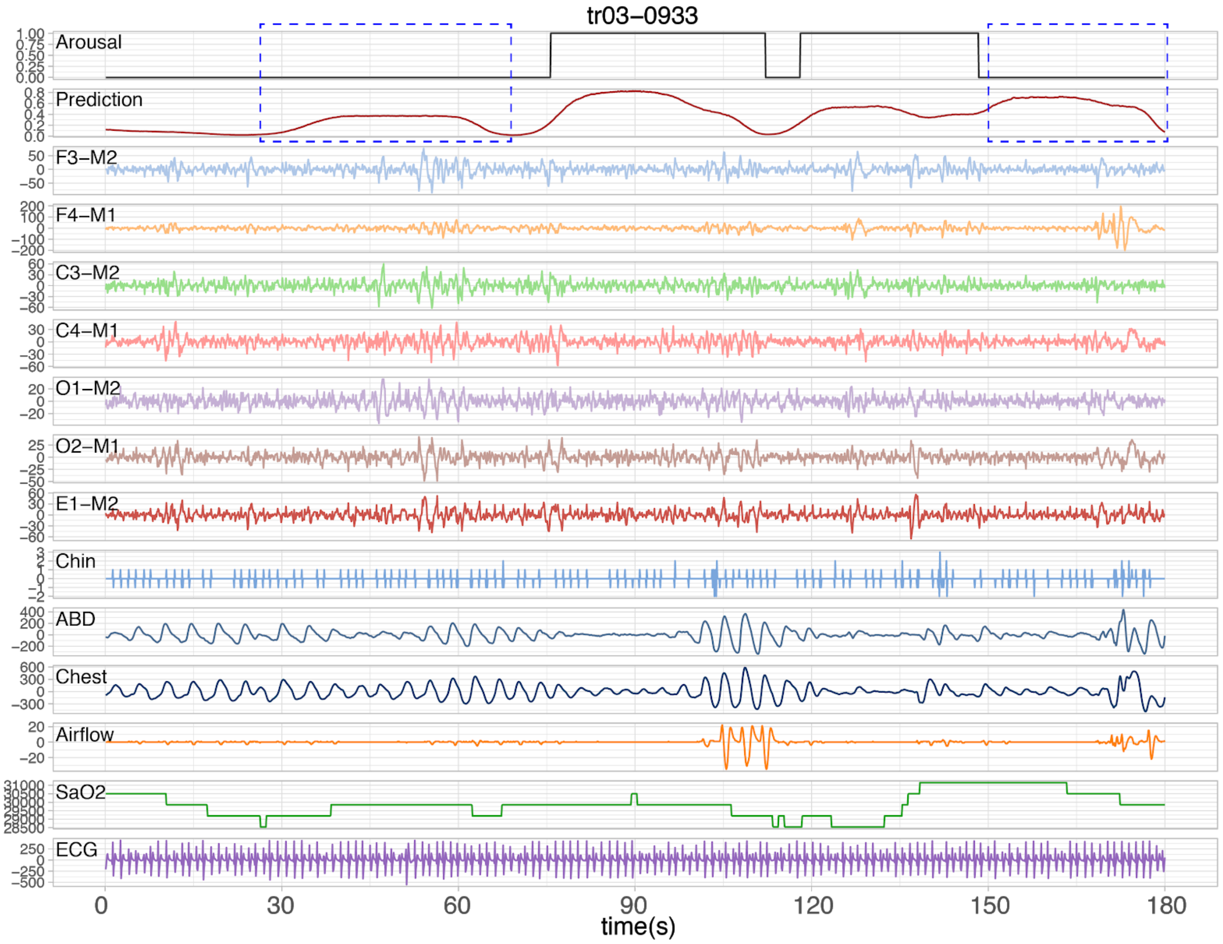

Supplementary Figure 9. A 180-second polysomnogram example with manual labels and predictions of sleep arousals.

From top to bottom, the sleep arousal labels (arousal = 1 and non-arousal=0), predictions by our algorithm, and 13-channel polysomnograms are shown for a total of 180 seconds (six 30-second epochs). Our algorithm detected suspected sleep arousal events shown in dashed blue rectangles.

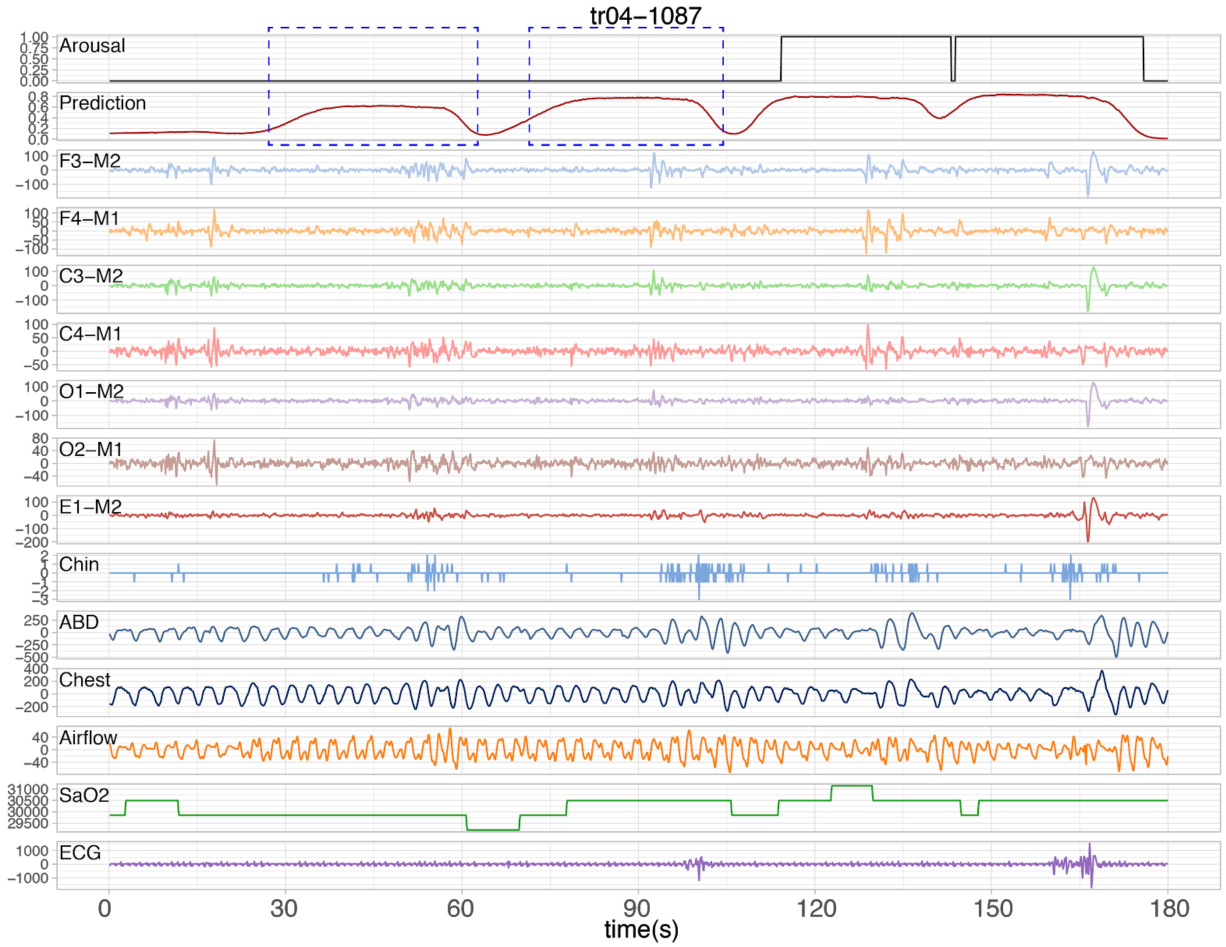

Supplementary Figure 10. A 180-second polysomnogram example with manual labels and predictions of sleep arousals.

From top to bottom, the sleep arousal labels (arousal = 1 and non-arousal=0), predictions by our algorithm, and 13-channel polysomnograms are shown for a total of 180 seconds (six 30-second epochs). Our algorithm detected suspected sleep arousal events shown in dashed blue rectangles.

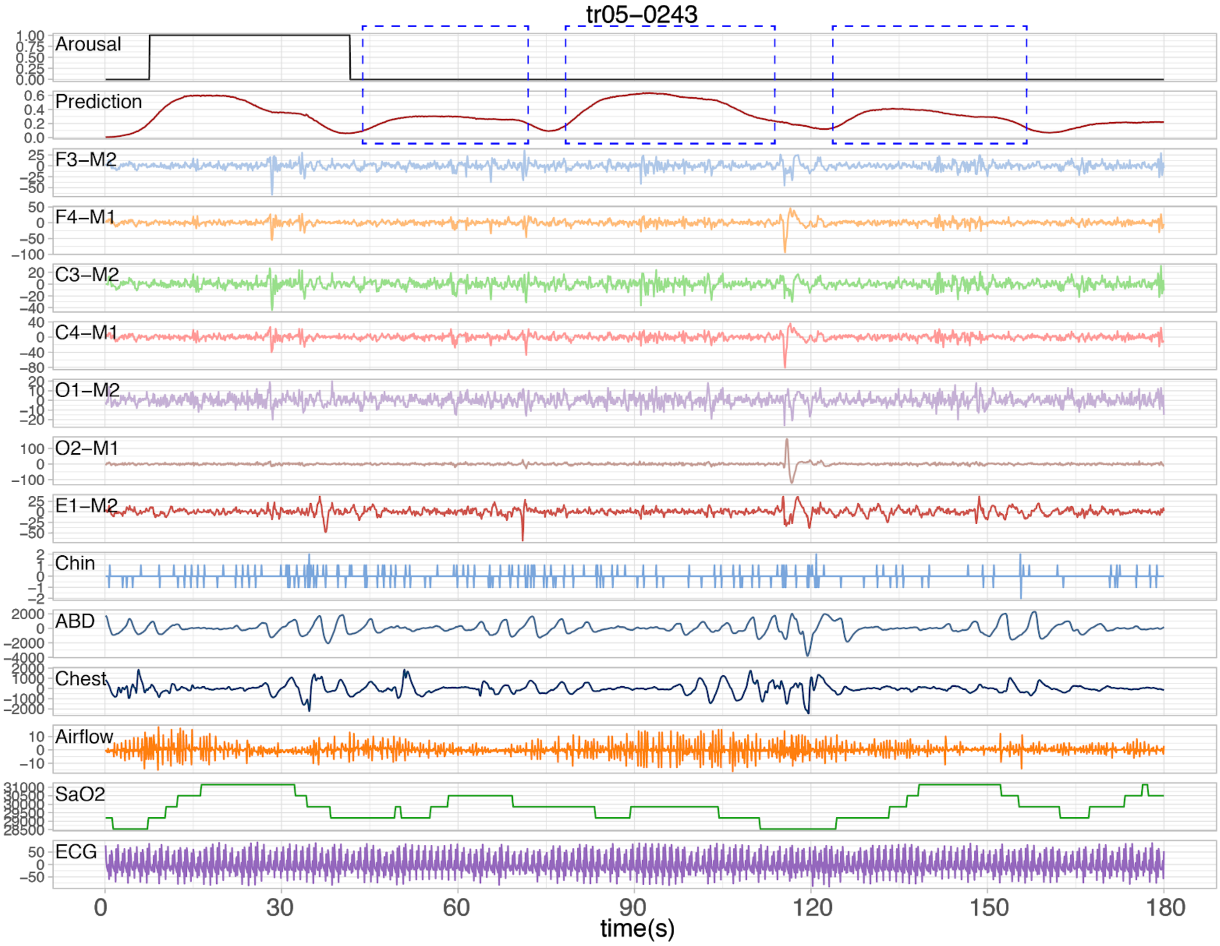

Supplementary Figure 11. A 180-second polysomnogram example with manual labels and predictions of sleep arousals.

From top to bottom, the sleep arousal labels (arousal = 1 and non-arousal=0), predictions by our algorithm, and 13-channel polysomnograms are shown for a total of 180 seconds (six 30-second epochs). Our algorithm detected suspected sleep arousal events shown in dashed blue rectangles.

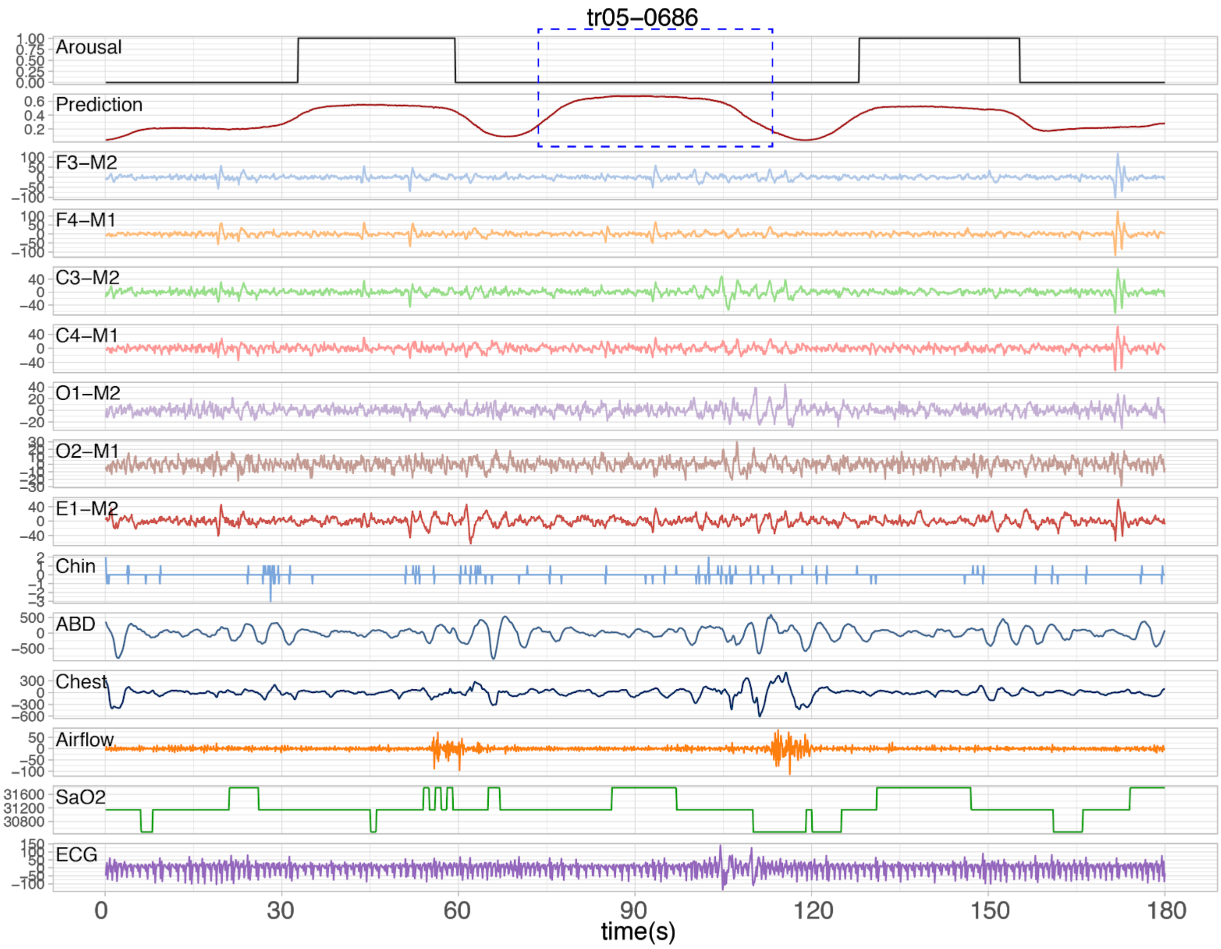

Supplementary Figure 12. A 180-second polysomnogram example with manual labels and predictions of sleep arousals.

From top to bottom, the sleep arousal labels (arousal = 1 and non-arousal=0), predictions by our algorithm, and 13-channel polysomnograms are shown for a total of 180 seconds (six 30-second epochs). Our algorithm detected a suspected sleep arousal event shown in the dashed blue rectangle.

Supplementary Table 1. The relationship between length of segments and the corresponding time.

length of segments(number of data points)	the corresponding time
2^23 = 8,388,608	41,943 seconds / 11.65 hours
2^22 = 4,194,304	20,972 seconds / 5.83 hours
2^21 = 2,097,152	10486 seconds / 2.91 hours
2^20 = 1,048,576	5,243 seconds / 1.46 hours
2^19 = 524,288	2,621 seconds / 43.7 minutes
2^18 = 262,144	1,311 seconds / 21.8 minutes
2^17 = 131,072	655 seconds / 10.9 minutes
2^16 = 65,536	328 seconds / 5.5 minutes
2^15 = 32,768	164 seconds / 2.7 minutes
2^14 = 16,384	82 seconds / 1.4 minutes
2^13 = 8,192	40.96 seconds
2^12 = 4,096	20.48 seconds
2^11 = 2,048	10.24 seconds
2^10 = 1,024	5.12 seconds
2^9 = 512	2.56 seconds
2^8 = 256	1.28 seconds

Supplementary Table 2. Clinical characteristics of the 2018 PhysioNet Challenge dataset

	total (n=1893)	train (n=994)	test (n=989)
gender (% male)	65	67	63
average age	55	55	55
drug use (%)			
antidepressant	26.1	25.7	26.5
antihistamine	4.8	4.8	4.8
benzodiazepine	16.1	16.9	15.4
hypertension	40.9	41	40.6
neuroleptic	4.2	4.5	3.8

opiate	7.4	8.1	6.7
neuroactive	19.1	20.8	17.5
sleep aids	28.3	29	27.8

Supplementary Table 3. Clinical characteristics of the SHHS Visit1 dataset

	total (n=1000)	train (n=750)	test (n=250)
gender (% male)	43.0	44.0	40.0
average age	72.8	72.9	72.5
race (%)			
white	85.4	85.5	85.2
black	11.5	12.1	9.6
others	3.1	2.4	5.2
disease (%)			
myocardial infarction	22.6	22.9	21.6
stroke	17.0	16.3	19.2
angina	18.1	19.3	14.4
congestive heart failure	23.8	23.3	25.2

Supplementary Table 4. Clinical characteristics of the SHHS Visit2 dataset

	total (n=1000)	train (n=750)	test (n=250)
gender (% male)	44.0	43.7	44.8
average age	67.9	68.2	66.9
race (%)			
white	86.1	85.3	88.4
black	11.0	11.6	9.2
others	2.9	3.1	2.4
disease (%)			
myocardial infarction	17.3	18.3	14.4
stroke	8.2	8.0	8.8
angina	31.2	30.4	33.6
congestive heart failure	16.3	16.5	15.6

Supplementary Table 5. Average AUPRC baseline and predictive performance for different gender and race groups

	baseline	prediction
gender		
male (Physionet)	0.077	0.509
female (Physionet)	0.061	0.475
male (SHHS1)	0.050	0.603
female (SHHS1)	0.040	0.598
male (SHHS2)	0.044	0.619
female (SHHS2)	0.032	0.600
race		
white (SHHS1)	0.044	0.601
black (SHHS1)	0.035	0.574
others (SHHS1)	0.065	0.627
white (SHHS2)	0.037	0.609
black (SHHS2)	0.045	0.585
others (SHHS2)	0.049	0.698

Supplementary References

1. Howe-Patterson, M., Pourbabaee, B. & Benard, F. Automated Detection of Sleep Arousals From Polysomnography Data Using a Dense Convolutional Neural Network. in *2018 Computing in Cardiology Conference (CinC)* vol. 45 (Computing in Cardiology, 2018).
2. Már Þráinsson, H. *et al.* Automatic Detection of Target Regions of Respiratory Effort-Related Arousals Using Recurrent Neural Networks. in *2018 Computing in Cardiology Conference (CinC)* vol. 45 (Computing in Cardiology, 2018).
3. He, R. *et al.* Identification of Arousals With Deep Neural Networks Using Different Physiological Signals. in *2018 Computing in Cardiology Conference (CinC)* vol. 45 (Computing in Cardiology,

2018).

4. Varga, B., Görög, M. & Hajas, P. Using Auxiliary Loss to Improve Sleep Arousal Detection With Neural Network. in *2018 Computing in Cardiology Conference (CinC)* vol. 45 (Computing in Cardiology, 2018).

5. Patane, A., Ghiasi, S., Pasquale Scilingo, E. & Kwiatkowska, M. Automated Recognition of Sleep Arousal Using Multimodal and Personalized Deep Ensembles of Neural Networks. in *2018 Computing in Cardiology Conference (CinC)* vol. 45 (Computing in Cardiology, 2018).

6. Miller, D., Ward, A. & Bambos, N. Automatic Sleep Arousal Identification From Physiological Waveforms Using Deep Learning. in *2018 Computing in Cardiology Conference (CinC)* vol. 45 (Computing in Cardiology, 2018).

7. Warrick, P. & Nabhan Homsi, M. Sleep Arousal Detection From Polysomnography Using the Scattering Transform and Recurrent Neural Networks. in *2018 Computing in Cardiology Conference (CinC)* vol. 45 (Computing in Cardiology, 2018).

8. Bhattacharjee, T. *et al.* SleepTight: Identifying Sleep Arousals Using Inter and Intra-Relation of Multimodal Signals. in *2018 Computing in Cardiology Conference (CinC)* vol. 45 (Computing in Cardiology, 2018).

9. Szalma, J., Bánhalmi, A. & Bilicki, V. Detection of Respiratory Effort-Related Arousals Using a Hidden Markov Model and Random Decision Forest. in *2018 Computing in Cardiology Conference (CinC)* vol. 45 (Computing in Cardiology, 2018).

10. Phan, H., Andreotti, F., Cooray, N., Chen, O. Y. & De Vos, M. Joint Classification and Prediction CNN Framework for Automatic Sleep Stage Classification. *IEEE Trans. Biomed. Eng.* (2018) doi:10.1109/TBME.2018.2872652.

11. Phan, H., Andreotti, F., Cooray, N., Chen, O. Y. & De Vos, M. SeqSleepNet: End-to-End Hierarchical Recurrent Neural Network for Sequence-to-Sequence Automatic Sleep Staging. *IEEE*

Trans. Neural Syst. Rehabil. Eng. (2019) doi:10.1109/TNSRE.2019.2896659.

12. Phan, H., Andreotti, F., Cooray, N., Chen, O. Y. & Vos, M. D. Automatic Sleep Stage Classification Using Single-Channel EEG: Learning Sequential Features with Attention-Based Recurrent Neural Networks. *Conf. Proc. IEEE Eng. Med. Biol. Soc.* **2018**, 1452–1455 (2018).
13. Biswal, S. *et al.* Expert-level sleep scoring with deep neural networks. *J. Am. Med. Inform. Assoc.* **25**, 1643–1650 (2018).
14. Sun, H. *et al.* Large-Scale Automated Sleep Staging. *Sleep* **40**, (2017).
